# Pre-contrast T1 mapping for detection of myocardial fibrosis in asymptomatic and symptomatic aortic stenosis

**DOI:** 10.1186/1532-429X-14-S1-P93

**Published:** 2012-02-01

**Authors:** Sacha Bull, Steven K White, Stefan K Piechnik, Andrew Flett, Vanessa Ferreira, Margaret Loudon, Jane M Francis, Stefan Neubauer, James Moon, Saul Myerson

**Affiliations:** 1 OCMR, Oxford, UK; 2 The Heart Hospital, London, UK

## Background

Aortic stenosis (AS) leads to diffuse fibrosis in the myocardium which may impair cardiac function. Existing techniques (late gadolinium enhancement [LGE]) are not good at detecting diffuse, as opposed to focal, fibrosis. Pre-contrast T1-mapping may identify changes in the myocardium without the need for exogenous contrast, and our aim was to investigate its ability to detect diffuse fibrosis in patients with AS.

## Methods

96 patients with moderate or severe AS were compared to 96 age-and gender-matched controls. Patients were categorized by severity of valve lesion (moderate: peak aortic velocity 3-4 m/s, severe: >4m/s) and symptoms. There were 80 asymptomatic patients under conservative management, and 16 patients with severe symptomatic AS awaiting valve replacement surgery (AVR). Biopsy samples for histological assessment of fibrosis were obtained in 11 of the latter group. All subjects underwent CMR at 1.5T which included pre-contrast T1-mapping using the Shortened Modified Look-Locker Inversion recovery (ShMOLLI) sequence. Average T1 values in the myocardium were analysed on a per-case basis.

## Results

**Figure 1:**
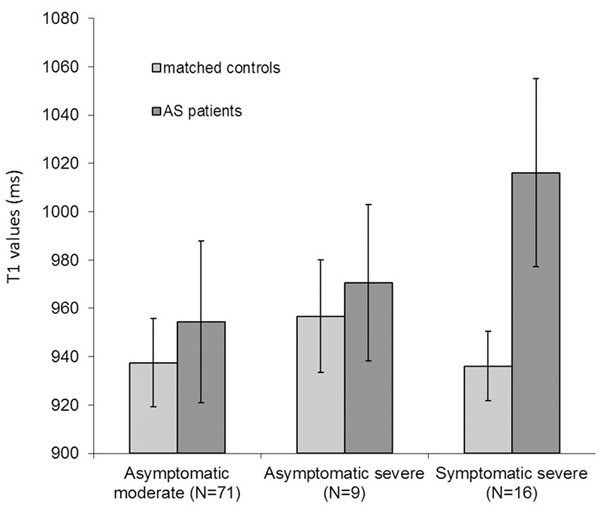
T1 values in normal controls and patients with aortic stenosis, categorised by severity of stenosis and symptoms.

**Figure 2:**
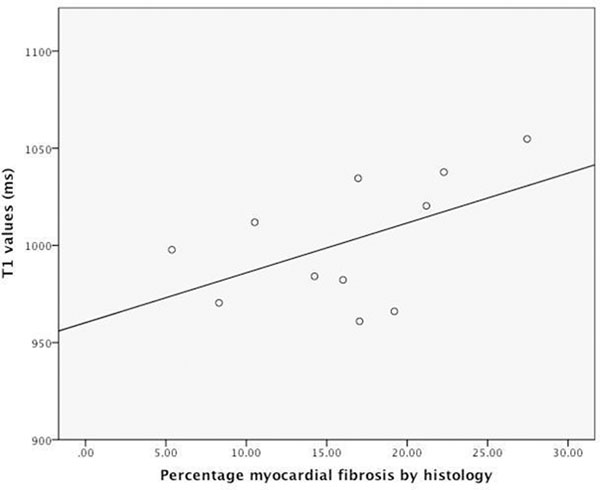
Correlation of myocardial T1 values with precent myocardial fibrosis by histological analysis.

The mean myocardial T1 value of all AS patients was longer than in controls (T1=966±41ms vs. 939±19ms respectively, P<0.0001). T1 values in the severe symptomatic patients were significantly longer than in asymptomatic patients (1016±39ms vs. 956±34ms respectively, p<0.003). There was no significant difference in mean T1 values between moderate (n=71) and severe (n=9) asymptomatic patients (p=0.3). When compared to matched controls, T1 values were significantly higher in the severe symptomatic group (T1=1016±39ms v 936±14 for controls, p<0.0001) and slightly higher for both asymptomatic groups (Figure 1; moderate: T1=954±33ms vs. 938±18ms for matched controls, p<0.02; severe: T1=971±32ms vs. 957±23, p=0.3). Comparison of the T1 values with histology showed a moderate correlation between the T1 value and percentage of fibrosis (r=0.51), though the sample size was small in this sub-set (n=11, Figure 2). There was also a reasonable correlation between T1 values and wall thickness (r=0.47). There was no significant difference in T1 values however according to the presence (n=48) or absence (n=23) of focal areas of LGE in the asymptomatic patients.

## Conclusions

Pre-contrast T1 values are increased in patients with AS compared to age- and gender-matched controls, particularly in the symptomatic patients, and these correlated with histological degrees of fibrosis. This suggests that pre-contrast T1 values may provide a useful assessment of diffuse myocardial fibrosis and may be a useful marker for monitoring AS progression.

## Funding

British Heart Foundation, Heart Research UK, Oxford Biomedical Research Centre, National Institute for Health Research.

